# APPLICATION OF POLYMERASE CHAIN REACTION (PCR) AND PCR BASED RESTRICTION FRAGMENT LENGTH POLYMORPHISM FOR DETECTION AND IDENTIFICATION OF DERMATOPHYTES FROM DERMATOLOGICAL SPECIMENS

**DOI:** 10.4103/0019-5154.39735

**Published:** 2008

**Authors:** R Bagyalakshmi, B Senthilvelan, K L Therese, S Murugusundram, H N Madhavan

**Affiliations:** *Larsen and Toubro Microbiology Research Centre, Vision Research Foundation, Sankara Nethralaya, No. 18, College Road, Chennai - 600 006, India*; 1 *Government Kilpauk Medical College, Kilpauk, Chennai - 600 010, India*; 2 *Dermatology Clinic (Skin, Hair and Nail Specialist), No. 2c, 2^nd^ Floor, No. 853, Thyagaraya Complex, Poonamalle High Road, Chennai, India*

**Keywords:** *18S rDNA*, *dermatophytes*, *PCR*, *PCR-RFLP*

## Abstract

**Objective:**

To develop and optimize polymerase chain reaction-based restriction fragment length polymorphism (PCR-RFLP) targeting 18S rDNA and internal transcribed spacer (ITS) region of fungi for rapid detection and identification of dermatophytes.

**Materials and Methods:**

Two PCR-RFLP methods targeting 18S rDNA and ITS regions of fungi were optimized using standard and laboratory isolates of dermatophytes and other fungi. Sixty-eight dermatological clinical specimens (nail clippings (56), material obtained from blisters (8), hair root (2), scraping from scaly plaque of foot (1) and skin scraping (1) collected by the dermatologist were subjected to both the optimized PCR-RFLP and conventional mycological (smear and culture) methods.

**Results:**

PCRs targeting 18S rDNA and the ITS region were sensitive to detect 10 picograms and 1 femtogram of *T. rubrum* DNA, respectively. PCR targeting 18S rDNA was specific for dermatophytes and subsequent RFLP identified them to species level. PCR-RFLP targeting the ITS region differentiated dermatophytes from other fungi with identification to species level. Among the 68 clinical specimens tested, both PCR-RFLP methods revealed the presence of dermatophytes in 27 cases (39.7%), whereas culture revealed the same only in 2 cases (7.40%), increasing the clinical sensitivity by 32.3%. Among 20 smear positive specimens, both PCR-RFLP methods detected dermatophytes in 12 (17.6%). Both the methods detected the presence of dermatophytes in 13 (19.11%) smear and culture negative specimens, increasing the clinical sensitivity by 36.1%.

**Conclusion:**

PCR-RFLP methods targeting 18S rDNA and the ITS regions of fungi were specific and highly sensitive for detection and speciation of dermatophytes.

## Introduction

Dermatophytes are a group of closely related fungi that invade the keratinized tissue (skin, hair and nails) of humans and other animals, resulting in an infection called dermatophytosis. These fungi are the commonest causes of superficial mycoses.^1^ Conventional laboratory methods based on detection of phenotypic characteristics such as microscopy and *in vitro* culture have played an essential role in dermatophyte identification. However, these procedures generally are either slow or nonspecific and the morphologic and physiologic characteristics depend on too many variables such as slow growth rate, presence of low threshold of organisms in clinical specimens, prior therapy and production of spores.^2^ Recent developments in the application of nucleic acid amplification technology have proved to enhance the quality of dermatophyte detection.^3^ Several nucleic acid-based molecular methods have been developed to detect fungi from clinical specimens targeting 18S rDNA,^4^,^5^ ITS1 and ITS2 regions^6^,^7^ 5.8S rDNA^8^ and 28S rDNA.^9^

Dermatophyte discrimination has met with some success using techniques such as polymerase chain reaction (PCR) targeting 18SrDNA,^10^ arbitrarily primed polymerase chain reaction (AP-PCR),^11^ random amplified polymorphic DNA analysis (RAPD),^12^ repetitive sequence PCR (rep-PCR),^13^ restriction analysis of mitochondrial DNA and ribosomal DNA.^14^,^15^ PCR for detection of dermatophytes have been widely employed targeting the nontranscribed spacer (NTS) regions, metalloprotease gene,^16^ chitin synthase (CHS) gene,^17^ tubulin gene, promoter region within ribosomal intergenic spacer, transcription elongation factor 1, actin gene and calmodulin gene.^18^ However, these approaches towards the speciation of the dermatophyte have not had significant success. Therefore, in the present study, two targets of the fungal genome - the ITS region and 18S rDNA - were chosen as they have cleavage sites that could be of value for application of RFLP on the amplified products not only to detect dermatophytes but also to speciation of the same in clinical specimens. PCR targeting 18S rDNA is known to be dermatophyte specific, and specific restriction enzyme sites are available in this region to differentiate the species using RFLP on amplified products.

## Materials and Methods

### Dermatophyte strains for standardization of the nucleic acid molecular biological and conventional mycological methods

Two standard strains comprising of *Trichophyton rubrum* ATCC 34265** and *Microsporum gypseum* ATCC 26652 supplied by P.G.I Chandigarh and 10 laboratory isolates containing *T. rubrum* (4)*, T. mentagrophytes* (3)*, M. gypseum* (2)** and *E. floccosum* (1) that were maintained in the laboratory were used in the study.

### Dermatological clinical specimens

Sixty-eight dermatological clinical specimens comprising nail clippings (56), material obtained from blisters (8), hair root (2), material obtained from scaly plaque of foot (1) and skin scraping (1) collected by the dermatologist were included in this study. The specimens collected in specially designed sterile paper envelope were transferred onto a sterile Petri plate in a clean air laminar flow work bench. A part of it was processed by conventional investigations and the other part for PCR methods.

### Conventional mycological investigations

The clinical specimens were processed for culture with subsequent identification carried out according to standard mycological methods.^1^ In brief, the specimens were inoculated onto Sabouraud's dextrose agar, Sabouraud's dextrose agar containing 16 μg/ml of chloramphenicol and 500 μg/ml of cycloheximide, potato dextrose agar and Trichophyton agar No.1 (HiMedia, India) and incubated at 25°C in a cooling incubator (Remi, Mumbai, India). All media were supplied in dehydrated form and prepared according to instructions of the manufacturer. Subculturing was done wherever necessary for further processes to identify the fungus. Fungal species were identified on the basis of culture characteristics, pigment production, microscopic examination in lactophenol cotton blue preparation and slide cultures. For direct microscopy, crushed smears of the specimen were prepared, 10% KOH-Calcofluor white wet mount was prepared and observed under fluorescence microscope with violet filter (Nikon, Japan) for the detection of fungi.

### Optimization of polymerase chain reaction

PCR on the specimens included the extraction of genomic DNA from dermatological specimens, followed by amplification using primers specific for 18S rDNA to detect dermatophytes and ITS primers for detection of fungal genome. PCR based RFLP using *Hae* III enzyme was applied on both the PCR amplicons to identify the species of dermatophytes and other fungi.

#### Fungal DNA extraction:

DNA from the isolates was extracted by following a modification of the Lee and Taylor protocol as described previously.^9^ The DNA from clinical samples was extracted following the Biogene Kit method (Biogene™ Corporals, USA). In brief, 200 μl (Optical density spectrophotometrically adjusted to 0.08 at 530 nm) of fungal isolate along with 3 μL proteinase K and 0.2 mL TBM™ buffer placed in pre-sterilized eppendorf vials was vortexed and incubated at 56°C for 30 min. After adding 0.2 mL of ethanol, it was spun at 8000 rpm for 1 min in the spin column provided in the kit. After decanting the filtrate and adding 0.5 mL of washing solution, it was spun again at 8000 rpm for 1 min and the filtrate was discarded. The third wash was done without washing the solution at 12000 rpm for 3 min. This step was followed by the addition of 0.1 mL of elution buffer and incubation at 56°C for 2 min. The DNA was recovered by spinning at 8000 rpm for 1 min and stored at −20°C.

### PCR Assay targeting 18SrDNA region using dermatophyte specific primers - DH1R and DH1L

Uniplex polymerase chain reaction (PCR) was carried out using primers DHIL (5' TGC ACT GGT CCG GCT GGG 3') and DH1R (5' CGG CGG TCC TAG AAA CCA AC 3') (5' ends at positions 631 and 813 according to the 18S rDNA sequence of *T. rubrum,* specific for dermatophytes,^10^ targeting the D2 subunit of the hypervariable V4 domain in the 18S rDNA region. The expected product length was 160-180 bp. The primers and PCR reagents were obtained from Bangalore Genei Pvt. Ltd, Bangalore, India. All PCR steps were carried out in a 50 μL reaction volume in 0.2 mL thin-wall polypropylene tubes (Axygen Inc., CA) using a Perkin-Elmer Thermal cycler (Model 2700). A 50 μL reaction with 200 μm concentration of each dNTP, 25 pmol of each primer, 1 U of *Taq* polymerase and 10 μL of template DNA. The PCR profile consisted of denaturation for 3 min at 95°C, followed by 35 cycles at 94°C for 1 min, 58°C for 1 min and 72°C for 40 sec and a final extension at 72°C for 5 min.

#### Analytical sensitivity:

Serial 10-fold aqueous dilution of standard strain of *T. rubrum* ATCC 34265 ranging from 10^−1^ to 10^−10^ was used to determine the analytical sensitivity.

#### Analytical specificity:

The specificity of the primers was tested with *T. rubrum, M. gypseum, E. floccosum, T. mentagrophytes*, standard strains of *Candida albicans* ATCC 24433, *C. tropicalis* ATCC750, *C. parapsilosis* ATCC 22019, *C. krusei* ATCC 6258 and lab isolates of *A. niger, A. terreus and Curvularia* spp.

### PCR assay targeting the ITS region

The ITS region was amplified applying two separate PCRs viz a seminested PCR to amplify ITS2, as described by Ferrer *et al*.^7^

### Application of PCR on dermatological specimens

A dermatophyte-specific uniplex PCR for amplifying ITS1 using primers as standardized and ITS PCR was applied on 68 dermatological specimens.

#### Detection of amplified products:

The products were visualized by running the products in a 2% agarose electrophoresis gel incorporated with 8 μL ethidium bromide at 100V for 20 min using a UV transilluminator (302n m) and documented using gel documentation system (Vilber Lourmat, France). Molecular weight markers (*Hinf* I digest of φX174 bacteriophage) were used in each run.

#### PCR-RFLP analysis:

The amplified products of dermatophyte-specific PCR were subjected to digestion with *Hae* III for 2 h and that of ITS amplicons were subjected to digestion with the same enzyme for 3 h. In a reaction volume of 25 μL containing 10 μL of PCR amplicons, 1 μL of *Hae* III (Bangalore Genei, India) and 2.5 μL of buffer c were added. The digested products were resolved using 4% agarose gel electrophoresis incorporated with 16 μL of ethidium bromide at 100 V and documented using (Vilber Lourmat, France)

## Results

Sensitivity and specificity of PCR assay targeting 18SrDNA (dermatophyte-specific PCR): The analytical sensitivity of PCR Assay targeting 18SrDNA region using dermatophyte specific primers-DH1R and DH1L was 10 picograms of ATCC strain of *T. rubrum*. The primers were specific, selectively amplifying all the dermatophyte isolates tested and not amplifying the other nondermatophye fungal DNA.

### Application of PCR on dermatological specimens

**Fig. 1:**
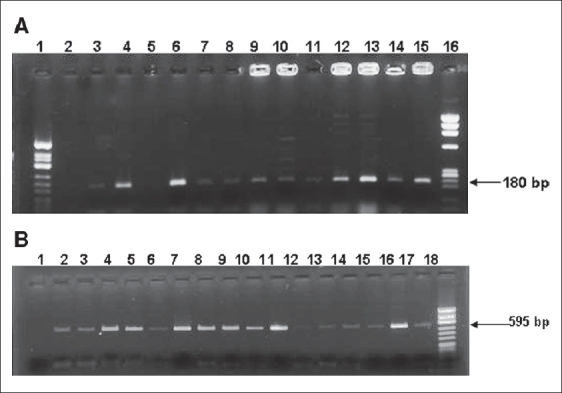
(A) Agarose gel electrophoresis showing the application of dermatophyte-specific PCR on dermatological specimens: Lane 1: Negative control, Lane 2: Nail clipping - Positive, Lane 3: Nail clipping - Positive, Lane 4: Nail clipping - Positive, Lane 5: Blister top - Positive, Lane 6: Blister top - Positive, Lane 7: Nail clipping- Positive, Lane 8: Nail clipping - Positive, Lane 9: Nail clipping - Positive, Lane 10: Nail clipping - Positive, Lane 11: Nail clipping - Positive, Lane 12: Nail clipping - Positive, Lane 13: Nail clipping - Positive, Lane 14: Positive control: standard strain of *T. rubrum*, Lane 15: Molecular weight marker *Hinf* – I digest of φX174 bacteriophage DNA; (B) Agarose gel electrophoresis showing the application of ITS PCR on dermatological specimens: Lane 1: Negative control, Lane 2: Nail clipping - Positive, Lane 3: Nail clipping - Positive, Lane 4: Nail clipping - Positive, Lane 5: Blister top - Positive, Lane 6: Blister top - Positive, Lane 7: Nail clipping - Positive, Lane 8: Nail clipping - Positive, Lane 9: Nail clipping - Positive, Lane 10: Nail clipping - Positive, Lane 11: Nail clipping - Positive, Lane 12: Nail clipping - Negative, Lane 13: Nail clipping - Positive, Lane 14: Nail clipping - positive, Lane 15: Blister top - Positive, Lane 16: Nail clipping - Positive, Lane 17: Positive control: *T. rubrum* standard strain DNA, Lane 18: Molecular weight marker – *Hae* - III φX174 bacteriophage DNA

The application of dermatophyte-specific PCR on dermatological specimens is shown in Fig. 1A and that of ITS PCR is shown in Fig. 1B.

### Application of PCR-RFLP using Hae III enzyme

**Fig. 2:**
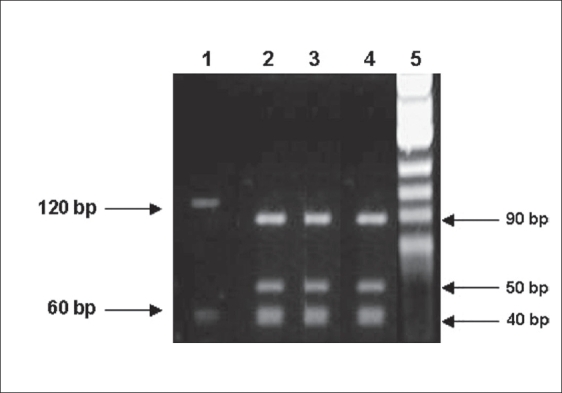
Agarose gel electrophoresis of PCR-RFLP performed on dermatophyte-specific PCR amplicons: Lane 1: Digested product of *M. gypseum* (120, 40 bp), Lane 2: Digested product of *T. rubrum* VRF 1478/06 (90, 50, 40 bp), Lane 3: Digested product of *T. rubrum* VRF 1593 /06 (90, 50, 40 bp), Lane 4: Digested product of *T. rubrum* standard strain, Lane 5: Molecular weight marker *Hinf*–I digest of φX174 bacteriophage

**Fig. 3:**
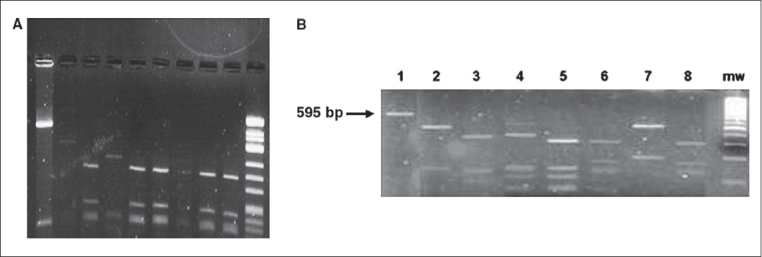
(A) Agarose gel electrophoresis of PCR-RFLP performed on ITS PCR amplicons to identify dermatophytes: Lane 1: Undigested product (595 bp), Lane 2: Digested product of *M. gypseum* (420, 95 bp), Lanes 3, 6, 7, 8 Digested product of T. rubrum (300, 200, 95 bp), Lane 4: Digested product of E. floccosum (350, 95 bp), Lane 9: Digested product of standard strain of T. rubrum, Lane 10: Molecular weight marker *Hinf* –I digest of φX174 bacteriophage DNA; (b) Agarose gel electrophoresis of PCR-RFLP performed on ITS PCR amplicons to identify nondermatophyte etiology: Lane 1: Undigested product *C. albicans* ATCC 24433 (595 bp), Lane 2: Digested product identified as *A. niger* (440, 150 bp), Lane 3: Digested product identified as *C. albicans* (331, 150, 100 bp), Lane 4: Digested product identified as *C. tropicalis* (351, 150, 100, 50 bp), Lane 5: Digested product identified as *C. parapsilosis* (331,150,100,50 bp), Lane 6: Digested product identified as *A. fumigatus* (301, 160,150 bp), Lane 7: Digested product identified as *A. flavus* (400, 195 bp), Lane 8: Digested product identified as *A. fumigatus* (301, 160, 150 bp), mw: Molecular weight marker *Hinf* –I digest of φX174 bacteriophage DNA

The results of application of PCR-RFLP using *Hae* III enzyme on dermatophyte-specific PCR and ITS PCR amplicons is shown in Figs. 2, 3A and 3B, respectively.

A comparison of the efficiencies of both conventional and PCR-based mycological methods was performed and the results are shown in the Table 1. Among the 68 specimens tested, 27 (39.7%) revealed the presence of dermatophytes by both methods of PCR-RFLP. Out of these 27 PCR positive specimens, 2 (7.40%) were culture positive, while PCR alone detected the presence of dermatophytes in 13 (19.11%) increasing the clinical sensitivity by 11.71%. All the specimens in which dermatophyte was detected by PCR targeting 18S RNA region were also positive by PCR for the ITS region. The RFLP on the amplified products of these 27 dermatophyte positive specimens identified the species as follows: *T. rubrum* in 21 specimens (Nail clipping 16 and blister top 5), *M. gypseum* in 5 specimens (nail clipping 4 and blister top 1) and *T. mentagrophytes* in 1 specimen (Hair root). In addition, PCR-RFLP on the ITS region detected the presence of DNA of nondermatophytes in 18 (26.4%) clinical specimens that were culture positive and 8 culture negative specimens, increasing the clinical sensitivity by 30.76%. Fungal DNA was not detected in 15 (22.05%) clinical specimens by both PCR methods and smear and cultures were also negative, indicating the specificities of both methods. Overall, the clinical sensitivity of PCR targeting 28s RNA increased by 31.67% over culture method. The fungal etiology associated with dermatomycoses is shown in Table 2: *A. flavus* - 5, *A. fumigatus* - 6, *A. niger* - 8, *Fusarium* species - 3, *A. terreus* - 1, 1 each of *C. albicans, C. tropicalis, C. parapsilosis*. *T. rubrum* (dermatophyte) and *A. niger* (nondermatophyte fungi) were found to be the principal etiological agents that caused dermatomycoses.

**Table 1:** Comparative analysis of the efficiency of smear, culture and PCR for the detection of dermatophytes in clinical specimens from the lesions of the patients clinically identified as dermatophytosis

PCR and PCR-RFLP identification *N* = 68	Smear positive culture positive clinical specimens speciation of fungi (*N* = 15)	Smear negative culture positive clinical specimens speciation of fungi (*N* = 5)	Smear positive culture negative clinical specimens speciation of fungi (*N* = 20)	Smear negative culture negative clinical specimens (*N* = 28)
PCR on 18S rRNA region Positive dermatophyte specific, *N* = 27 (39.07%)@	2 (2.9%)*	Nil	12 (17.64%)@	13 (19.11%)@
	*T. rubrum* 1		*T. rubrum* 9	*T. rubrum* 11
	*M. gypseum* 1		*M. gypseum* 3	*M. gypseum* 1
PCR-RFLP species identification*				*T. mentagrophytes* 1
PCR-RFLP on ITS region#	15 (22.05%)	Nil	20 (29.41%)	13^δ^ (19.11%) dermatophytes
positive specific for fungi including dermatophytes,	2^£^ (2.9%) dermatophytes;		12° (17. 64%) dermatophytes;	
*N* = 53 (77.9%)	13 (19.11%)*	5 (7. 35%)	8 (11.76%)	Nil
	nondermatophyte fungi:	Nondermatophyte fungi:	nondermatophyte fungi:	
	*A. niger* 4	*A. niger* 3	*A. flavus* 3	
	*A. flavus* 2	*A. fumigatus* 1	*A. fumigatus* 2	
	*A. fumigatus* 3	*Fusarium sp* 1	*A. niger* 1	
	*C. albicans* 1		*A. terreus* 1	
	*C. tropicalis* 1		*Fusarium sp* 1	
	C. parapsilosis 1			

* Results indicate 100% specificity of PCR on 18S rRNA region of the fungus to detect dermatophyte in clinical specimens

@ Clinical sensitivity increased by (17. 64% + 19.11%) 31.67% over conventional methods of smear and culture; Statistically signiT cant by Fisher Exact test p value > 0.0001

# PCR on ITS region is panfungal genome specific; it detects fungi, including dermatophytes; 2^£^Results of PCR-RFLP on ITS region identified the dermatophytes as those of PCR-RFLP on 18S rRNA region; 12°Results of PCR-RFLP on ITS region identified the dermatophytes as those of PCR-RFLP on 18S rRNA region; 13^δ^Results of PCR-RFLP on ITS region identified the dermatophytes as those of PCR-RFLP on 18S rRNA region

**Table 2:** Fungi identified from dermatological specimens obtained from 68 patients with dermatomycoses

Clinical diagnosis and dermatological specimens (68)	Fungal etiology associated with dermatological lesions		

	*Dermatophyte fungi identified*	*Nondermatophyte fungi identified*	PCR and culture negative
**Nail clipping (56)**			
Total onycholysis following trauma (4)	*T. rubrum* 1, *M. gypseum* 1*	*C. albicans* 1#	1
Onychomycosis (29)	*T. rubrum* 8*, *M. gypseum* 3	*A. niger* 6#@$, *A. flavus* 1#	6
		*A. terreus* 1$, *C. tropicalis* 1#	
		*A. fumigatus* 1#, *Fusarium*	
		species 2#$	
Diabetic distal onycholysis (2)	*T. rubrum* 2	-	-
Traumatic onychomycosis (3)	*T. rubrum* 2	*C. parapsilosis* 1#	-
Chronic paraonychia with onychomycosis (2)	*T. rubrum* 1	*A. flavus* 1#	-
Distal nail dystrophy (2)	-	*A. flavus* 2$	-
Subungual hyperkeratosis (1)	*T. rubrum* 1	-	-
Ingrown nail (5)	*T. rubrum* 1	*A. niger* 1#, *A. fumigatus* 2#	1
Psoriasis of the nail (3)	*T. rubrum* 1	*A. fumigatus* 2$	-
Lichen planus of nail (3)	*T. rubrum* 1	*A. niger* 1#	1
Idiopathic Nail dystrophy (2)	-	*A. fumigatus* 1@	1
**Blisters top (8)**			
Pompholyx (vesicular eczema) (2)	*T. rubrum* 1	*Fusarium* species 1@	-
Onycholysis with renal insufficiency (1)	*M. gypseum* 1	-	-
Onychomycosis (4)	*T. rubrum* 1	*A. flavus* 1	2
Onychomycosis and brittle nail (1)	-	-	1
**Others (4)**	-	-	1
Tinea versicolor (2)	*T. rubrum* 1 (Skin scraping)		1
Depigmented hair (2)	*T. mentagrophytes* 1 (Hair root)		1

* *T. rubrum* (1) and *M. gypseum* (1) were isolated in culture and identified by dermatophyte specific PCR-RFLP and PCR-RFLP on the ITS region, thereby yielding concordant results; The other dermatophyte fungi were identified by dermatophyte-specific PCR-RFLP and PCR-RFLP on the ITS region

# The nondermatophyte fungi were detected in smear, isolated in culture and identified by PCR-RFLP on the ITS region. The results of PCR-RFLP were concordant with that of conventional identification

@ The nondermatophyte fungi were isolated in culture (not detected in direct smear) and identified by PCR-RFLP on the ITS region. The results of PCR-RFLP were concordant with that of conventional identification

$ The nondermatophyte fungi were detected in smear and identified by PCR-RFLP on the ITS region

## Discussion

This study was designed to develop PCRs for rapid detection and species level identification of dermatophytes and other fungi that cause dermatomycoses. The results clearly demonstrated not only the specificity but also an increased clinical sensitivity by 36.1% and reliable rapid results within 24 h in contrast to the 21 days of incubation required for the isolation of dermatophytes by culture.

PCR targeting the ITS region was considered as the gold standard in this study as application of this PCR indicated the presence of fungus in 77.9% specimens out of which 39.4% were dermatophytes and the rest (38.5%) were nondermatophytes. The superiority of the ITS PCR was also reflected in detection of more number of nondermatophyte fungi involved in causing dermatomycoses and its specificity in proving 15 of the 68 clinical specimens negative for fungal etiology, which were also negative by smear culture and also by dermatophyte specific PCR. In addition, ITS PCR-based RFLP proved to be a confirmatory technique for dermatophyte-specific PCR targeting 18SrDNA gene in detection and identification of the species of dermatophytes with 100% correlation. Based on these factual findings, we consider ITS PCR as the gold standard for this study.

In the literature, the percentage of dermatophytes isolated by conventional methods range from 30% to 70% in the specimens from clinically diagnosed dermatophytosis patients.^19^–^24^ However, in the present study, the culture positivity for dermatophytes was low (2.94%) in spite of using special media such as Trichophyton agar and Dermatophyte medium incorporated with cycloheximide and chloramphenicol and incubation for 30 days. The low yield of culture in the present study could be attributed to low threshold of organisms and/or prior antifungal therapy. The clinical specificity of the dermatophyte PCR also was clearly demonstrated by the fact that none of the 26 nondermatophyte fungi (18 identified by culture and 8 positive by ITS PCR) were detected by dermatophyte-specific PCR. Generally, nondermatophytes are believed to constitute approximately 10% of the causative agents of onychomycosis.^25^ The nondermatophyte fungi are quite common in the hot and humid tropical countries such as India,^26^,^27^ unlike the West where the nondermatophyte moulds and yeasts are found as contaminating organisms in dermatophyte onychomycosis, secondary to dermatophytosis. Elewski *et al*.^28^,^29^ have reported *Aspergillus, Candida* and *Fusarium* species as the nondermatophytes that cause dermatomycoses, correlating well with the present study.

PCR-RFLP has revolutionized diagnostic microbiology and it helps to arrive at a more specific diagnosis by reducing the time, labor and poor isolation rate of conventional culture methods In a diagnostic set up both ITS PCR and dermatophyte-specific PCR can be applied on dermatological specimens since any fungi causing dermatomycoses can be detected. Thus, this study demonstrates the superiority of PCR-based techniques in terms of sensitivity rapidity and reliability when compared to the conventional mycological culture method.
